# Aortic aneurysm: Not an obstacle for refractory summit ventricular tachycardia

**DOI:** 10.1016/j.hrcr.2025.10.010

**Published:** 2025-10-17

**Authors:** Sara Vázquez-Calvo, Pasquale Valerio Falzone, Frida Eulogio-Valenzuela, Andreu Porta-Sánchez, Ivo Roca-Luque

**Affiliations:** 1 Institut Clinic Cardiovascular, Hospital Clínic, Universitat de Barcelona, Catalonia, Spain; 2 Institut d’Investigacions Biomèdiques August Pi i Sunyer (IDIBAPS), Barcelona, Catalonia, Spain; 3 Centro de Investigación Biomédica en Red de Enfermedades Cardiovasculares (CIBERCV), Madrid, Spain

**Keywords:** Summit region, Ventricular tachycardia, Catheter ablation, Imaging, Aortic aneurysm, Refractory ventricular tachycardia, Transseptal puncture

**Key Teaching Points**

▪ Left ventricular summit arrhythmia exhibits specific electrocardiographic characteristics, primarily right bundle branch block in precordial leads or left bundle branch block with transition in V2 or V3 or pattern break in V2.
▪ The presence of cardiomyopathy should always be ruled out.
▪ Catheter ablation is a highly effective procedure, particularly indicated in symptomatic cases refractory to medical treatment; however, this location can pose challenges.
▪ Transseptal access for left ventricular summit arrhythmias is increasingly preferred over retrograde aortic approach, given that it may reduce procedural risks and can be safely performed even in patients with complex anatomy, when guided by imaging.

## Introduction

Ventricular tachycardia (VT) originating from the left ventricular summit (LVS) represents a therapeutic challenge owing to the complex anatomy of this region and its proximity to major coronary arteries. Although antiarrhythmic therapy can be attempted initially, these arrhythmias often prove refractory, making catheter ablation the most effective option for symptom control and arrhythmia suppression. However, access to the LVS can be technically demanding, particularly in patients with structural or anatomic constraints.

This report describes the case of an elderly patient with recurrent syncopal episodes caused by drug-refractory monomorphic VT arising from the LVS, in whom the presence of a large ascending aortic aneurysm precluded a retrograde approach. The case highlights the feasibility and safety of a transseptal, imaging-guided ablation strategy under such challenging conditions.

## Case report

An 87-year-old patient presented with a 3-year history of recurrent syncopal episodes occurring both at rest and during physical activity, without identifiable triggers or prodromal symptoms. These episodes frequently resulted in significant trauma, severely affecting the patient’s quality of life.

The patient’s medical history was notable for valvular heart disease, requiring biological aortic valve replacement 15 years prior. Additional comorbidities included dyslipidemia, arterial hypertension, chronic kidney disease, and a chronic ascending aortic aneurysm deemed unsuitable for surgical intervention owing to high surgical risk. Serial computed tomography (CT) scans demonstrated progressive aneurysmal growth.

**Figure 1:**
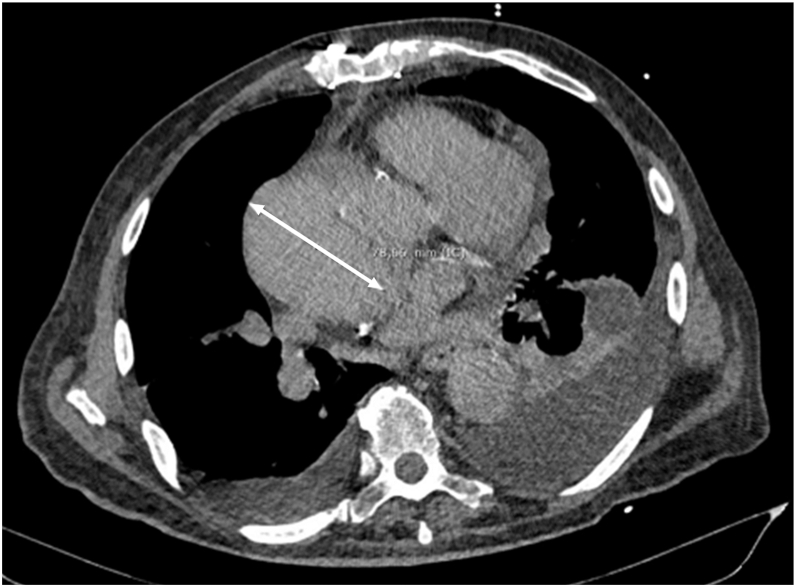
Transaxial computed tomography scan image showing an ascending aortic aneurysm of 7.8 cm.

**Figure 2:**
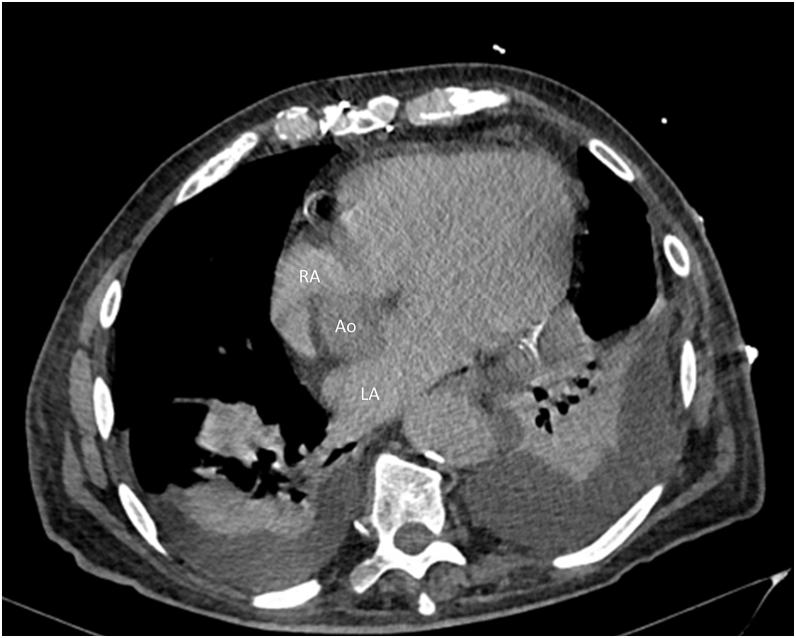
Location of the aortic aneurysm between the 2 atria, potentially complicating transseptal punction. Ao = aortic aneurysm; LA = left atrium; RA = right atrium.

Initial laboratory investigations were unremarkable. Transthoracic echocardiography confirmed normal prosthetic valve function and preserved left ventricular ejection fraction, ruling out significant structural heart disease. During a syncopal episode, an electrocardiogram (ECG) recorded VT with a positive QRS complex in all precordial leads and an inferior axis, suggestive of an origin in the LVS. Cardiac CT further identified a partially thrombosed ascending aortic aneurysm measuring 7.8 cm in diameter, displacing the right atrium posterolaterally (Figures 1 and 2).

Antiarrhythmic therapy with amiodarone and bisoprolol was initiated but failed to control the VT episodes. The patient continued to experience frequent syncopal events, some leading to cranioencephalic trauma. Alternative pharmacologic approaches, including calcium channel blockers, mexiletine, and sotalol, were subsequently trialed in sequence owing to the insufficient efficacy of previous therapies. Given the refractory nature of the VT and despite the elevated risk of aortic aneurysm rupture, catheter ablation was pursued.

The procedure was performed under general anesthesia, with continuous hemodynamic monitoring, including a radial arterial line. VT induction was achieved via right ventricular apical pacing. ECG findings during VT induction demonstrated a right bundle branch block (RBBB) morphology with an inferior axis, a longer R wave in lead III than in lead II, a pseudo-delta wave, and a negative deflection in lead I, consistent with a summit region origin. Mapping within the coronary sinus and great cardiac vein was attempted, but electrograms in these locations showed poor precocity and were not suitable as ablation targets, prompting left ventricular mapping.

A transseptal puncture was performed using a Brockenbrough needle and SL1 (Abbott Medical) sheath under transesophageal echocardiographic guidance. To minimize the risk of aneurysm puncture, the puncture site was deliberately selected in the lowest region of the interatrial septum. The SL1 sheath was then exchanged for a deflectable sheath (Agilis, Abbott Medical). Particular care was taken to avoid inadvertent crossing of the aortic valve or interaction with the aneurysmal ascending aorta. Standard intraprocedural anticoagulation with intravenous unfractionated heparin was administered after transseptal puncture (target activated clotting time >300 seconds) to mitigate thromboembolic risk.

**Figure 3:**
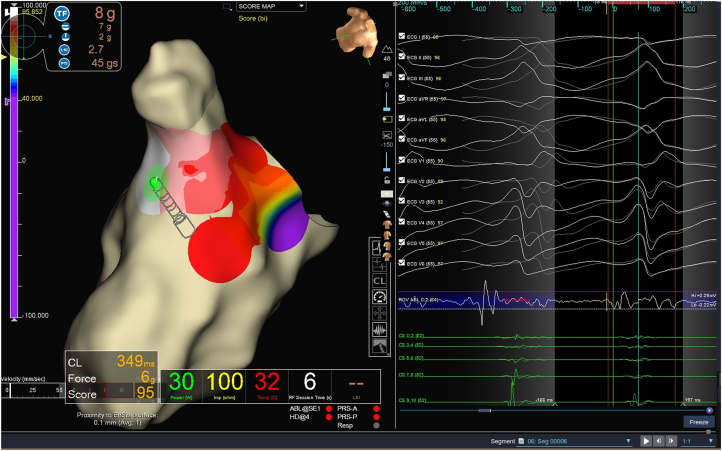
Electroanatomic mapping during ventricular tachycardia ablation. A pace map of 95% is observed in the anteroseptal subaortic valve region with precocity of −35 ms.

**Figure 4:**
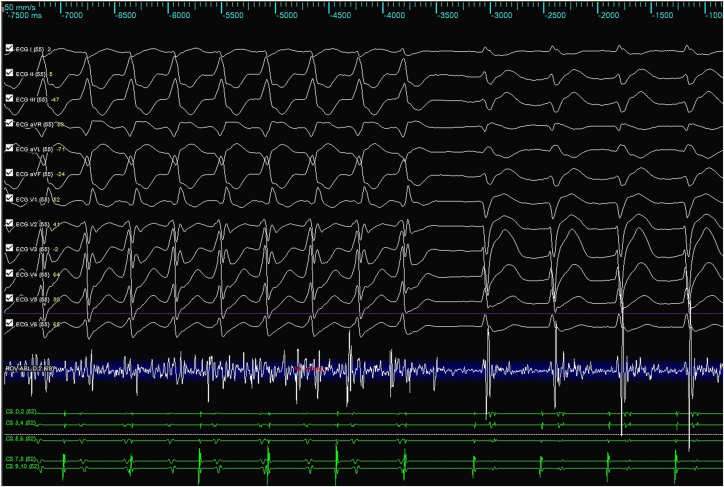
Ventricular tachycardia stopped during ablation in this location.

Activation mapping with an HD Grid catheter (Abbott Medical) and the Ensite X System (Abbott Medical) identified the earliest activation site in the left ventricular outflow tract, specifically in the anteroseptal region just below the aortic valve. The local electrogram preceded the QRS onset by 35 ms, and pace mapping at this site demonstrated a 95% match to the clinical VT morphology (Figure 3). Radiofrequency (RF) ablation was performed using a TactiCath (Abbott Medical) catheter (45 °C, power limit of 40 W, irrigation flow 26–30 mL/min), resulting in VT termination within 3 seconds of energy delivery (Figure 4). Lesions were impedance guided, with a duration ranging between 40 and 60 seconds and a minimum contact force of ≥7 g, and the target area was reinforced with 3 additional surrounding applications. Postablation induction protocols failed to reinduce VT, and the procedure was completed without immediate complications.

## Discussion

The LVS is a triangular area located at the most superior portion of the left epicardial ventricular region, surrounded by the 2 branches of the left coronary artery: the left anterior interventricular artery and the left circumflex artery. Arrhythmias originating from the LVS constitute 14% of all left ventricular arrhythmias.^1^

The most common ECG abnormalities in LVS arrhythmia include RBBB or left bundle branch block with V2 or V3 transition, a longer R wave in III than in II, a pseudo-delta wave and/or maximum deflection index of 0.55, a V2 pattern break, a negative lead I, and a more negative aVL than aVR.^1^ Several ECG patterns have been described to predict ablation success or the potential best target location. For instance, the RBBB pattern is typical of PVCs originating from the accessible zone (usually through the great cardiac vein).^2^

Clinical guidelines^3^ recommend excluding underlying structural heart disease in cases of VT. For idiopathic VT, when patients are symptomatic or associated with deterioration of cardiac function, ablation is considered as the first-line therapy for right ventricular outflow tract and fascicular VT. Antiarrhythmic drugs can be chosen as a first option in patients with summit arrhythmia owing to the lowest overall ablation success (70%)^4^ and a higher risk of complications than other locations, such as the right ventricular outflow tract. On the one hand, accessing the epicardium is complicated by nearby coronary vessels and intervening epicardial fat.^2^ Although certain portions of the summit area may be reached through the great cardiac vein, the effectiveness of RF ablation lesions is often restricted by the high impedance encountered in smaller veins.^4^ On the other hand, the nearest endocardial surfaces may be too distant from the VT origin, rendering conventional endocardial RF ablation less effective.^5^ Notably, endocardial procedures to ablate LVS arrhythmias tend to achieve success more frequently in the left ventricular outflow tract, followed by the aortic cusps, and seldom in the right ventricular outflow tract. Several strategies have been formulated to increase the depth of the lesion in case of initial failure applications, with the most commonly used method being an extension of the RF duration. This approach has been demonstrated to be a safe and efficient technique.^6^

Considering the frequent need to perform ablation lesions from the aortic cusps or the subaortic region, retroaortic access is chosen by most centers.^7^ This approach circumvents the difficulty of reaching the aortic cusp after a transseptal puncture, which would involve traversing a greater distance across the right atrium, left atrium, and left ventricle before finally reaching the intended ablation point, usually needing a reversed S curve of the ablation catheter.^8^ Nevertheless, the recently published TRAVERSE study^9^ has demonstrated that transseptal access for left ventricular ablation reduces the risk of acute brain lesions compared with a retrograde aortic approach, without compromising procedural safety or efficacy. In cases of aortic aneurysm or other extrinsic factors affecting transseptal anatomy, careful imaging-guided transseptal puncture^10^ can further mitigate procedural risks.

Beyond conventional manual catheter navigation, other strategies such as remote magnetic navigation (Stereotaxis system) or stereotactic radiotherapy have also been explored for the management of arrhythmias arising from anatomically complex regions like the LVS. Remote magnetic navigation enables highly precise and stable catheter movement, facilitating navigation within technically demanding areas—such as the LVS—with fine control of catheter orientation and contact force, and can be effectively used both via retroaortic^11^ and transseptal approaches,^12^ where manual maneuvering would otherwise be difficult. Similarly, stereotactic radiotherapy has been reported as a feasible and effective alternative for treating refractory ventricular arrhythmias, including cases involving the summit region.^13^

To the best of our knowledge, this is the first case report of a successful VT ablation from the summit region using a transseptal access in a patient with an enormous ascending aortic aneurysm.

## Follow-up

A control CT scan was performed 1 month later showing an increase in the size of the aneurysm, from 7.8 to 9.4 cm, likely reflecting the natural rapid progression of a giant ascending aortic aneurysm. One year later, the patient remains alive and asymptomatic for cardiovascular symptoms. Given the patient’s advanced age and very frequent syncopal episodes before ablation, follow-up was based exclusively on clinical evaluation, with the primary goal of improving quality of life rather than pursuing detection of every subclinical arrhythmia.

## Conclusion

Arrhythmia from the LVS region can be refractory to several schemes of medical treatment, with catheter ablation being an effective treatment. Ablating LVS arrhythmia can pose challenges owing to its epicardial origin, requiring the meticulous mapping of various structures surrounding the foci. In this sense, an aortic aneurysm can be an obstacle by impeding retroaortic access for an aortic cusp ablation. A transseptal access can be an alternative approach enabling the ablation target to be reached in very symptomatic patients with refractory VT.

## Disclosures

I.R.L. and A.P.S. report activities as consultants and lecturers for Biosense Webster, Medtronic, Boston Scientific, and Abbott Medical. All other authors report no relationships relevant to the contents of this paper to disclose.
